# BMP2 Is Related to Hirschsprung’s Disease and Required for Enteric Nervous System Development

**DOI:** 10.3389/fncel.2019.00523

**Published:** 2019-12-03

**Authors:** Sizhou Huang, Yi Wang, Lingfei Luo, Xiaoqing Li, Xianqing Jin, Shuangshuang Li, Xiaoping Yu, Min Yang, Zhenhua Guo

**Affiliations:** ^1^Development and Regeneration Key Laboratory of Sichuan Province, Department of Anatomy and Histology and Embryology, Chengdu Medical College, Chengdu, China; ^2^Ministry of Education Key Laboratory of Child Development and Disorders, Children’s Hospital of Chongqing Medical University, Chongqing, China; ^3^Key Laboratory of Pediatrics in Chongqing, CSTC2009CA5002, Children’s Hospital of Chongqing Medical University, Chongqing, China; ^4^Chongqing International Science and Technology Cooperation Center for Child Development and Disorders, Children’s Hospital of Chongqing Medical University, Chongqing, China; ^5^Key Laboratory of Aquatic Organism Reproduction and Development, Ministry of Education, School of Life Sciences, Southwest University, Chongqing, China; ^6^Key Laboratory of Aquatic Science of Chongqing, School of Life Sciences, Southwest University, Chongqing, China; ^7^Laboratory of Molecular Developmental Biology, School of Life Sciences, Southwest University, Chongqing, China; ^8^Department of Public Health, Chengdu Medical College, Chengdu, China; ^9^School of Laboratory Medicine, Chengdu Medical College, Chengdu, China

**Keywords:** BMP2, GDNF, Hirschsprung’s disease, enteric nervous system, development

## Abstract

The enteric nervous system (ENS) is derived from neural crest cells (NCCs). Defects in ENS NCCs colonizing in the intestines lead to an absence of enteric ganglia in the colon and results in Hirschsprung’s disease (HSCR). Bone morphogenetic proteins (BMPs) play diverse roles in the proliferation, migration and survival of ENS NCCs; however, whether BMPs are involved in HSCR and the underlying mechanism remains largely unknown. In this study, we found that BMP2 expression is significantly decreased in HSCR patients. Further experiments demonstrated that BMP2 is involved in the regulation of NCC proliferation, migration and differentiation. In a detailed analysis of the role of BMP2 in HSCR development *in vivo*, we demonstrated that BMP2b regulates the proliferation, migration and differentiation of vagal NCCs in zebrafish and that BMP2b is required for intestinal smooth muscle development. In addition, we showed that BMP2b is involved in regulating the expression of glial cell line-derived neurotrophic factor (GDNF) in the intestine, which mediates the regulation of ENS development by BMP2b in zebrafish. These results highlight a central role of the BMP-GDNF cascade in intestinal patterning and ENS development. Our results further demonstrate the key role of BMP2 in the etiology of HSCR *in vitro* and *in vivo*.

## Introduction

Hirschsprung’s disease is a common congenital disorder with an incidence of 1 in 5000 live births. This condition results from disordered motor function of the distal alimentary tract, including the internal anal sphincter, and an absence of parasympathetic intramural ganglion cells in the hindgut, which leads to intestinal obstruction in neonates and intractable constipation in infants and adults (Brooks et al., 2005; Emison et al., 2005; Zundel et al., 2010). HSCR is related to the obstruction of ENS development. The ENS, which is derived from the neural crest, is the largest subdivision of the PNS (Lake and Heuckeroth, 2013). In vertebrates, the ENS is derived from NCCs (Heanue and Pachnis, 2007), which are pluripotent cells capable of migrating and forming a wide variety of neuronal subtypes and enteric glia (Lake and Heuckeroth, 2013). The migration of ENS precursors during intestinal development is strongly dependent on mutual signaling interactions between the gut endoderm and precursors.

The molecular mechanisms that control ENS precursor development have been partly studied *in vivo* and *in vitro*. Various transcription factors and the receptor tyrosine kinase family, including RET (Edery et al., 1994), SOX10 (Pelet et al., 1998), Phox2b (Pattyn et al., 1999), and Hand2 (D’Autreaux et al., 2007), are involved in ENS development. Moreover, several secreted ligands and their receptors control the migration, proliferation and differentiation of NCCs either directly or indirectly, including GDNF (Moore et al., 1996), EDN3 (Baynash et al., 1994), Sonic hedgehog (Reichenbach et al., 2008), CNTF (Chalazonitis et al., 1998), and Neurturin (Heuckeroth et al., 1998). Dysfunction of these transcription factors and secreted signaling molecules/receptors results in defects in the ENS. Furthermore, mutations in some of these genes have been confirmed in HSCR patients (Amiel et al., 2007). BMPs regulate multiple critical functions in embryonic development, including organ morphogenesis; epithelial-mesenchymal interactions; and cell migration, proliferation and differentiation within the developing gut (Smith et al., 2000). In vertebrates, BMPs are strongly expressed in the gut epithelium adjacent to the mesenchyme (Goldstein et al., 2005). More recently, it has been reported that the number of neurons developing from enteric crest-derived cells is increased by low concentrations and decreased by high concentrations of BMP-2 or BMP-4 (Chalazonitis, 2004). These findings are significant given that BMPs have concentration-dependent effects on the induction of the neuronal differentiation of enteric neural crest stem cells and affect the differentiation of ENS precursors. In addition, treatment of gut explants with noggin (an antagonist of BMP2 and BMP4) reduces neural crest-derived cell migration from the gut explants toward sources of GDNF and delays ENS precursor migration to the distal bowel in chicks (Goldstein et al., 2005). Contradictory to these findings, noggin was found to increase NCC migration in murine experiments (Fu et al., 2006). The different effects of noggin on NCC migration between chicks and mice may be because the colonization of the myenteric plexus region by NCCs occurs first in the differentiated gut (Fu et al., 2006). Although the role of BMPs in ENS development have been reported, the underlying mechanism remains unknown. In addition, whether aberrant BMP activity is involved in the regulation of ENS defects in patients is largely unknown.

Here, we demonstrated that BMP signaling activity was significantly reduced in HSCR patients, and *in vitro* experiments demonstrated that BMP2 promotes the proliferation, migration and differentiation of NCCs. To further study the role of BMPs in ENS development among HSCR patients, we utilized zebrafish (*Danio rerio*) to determine the role of BMP2b in ENS development in detail. Our results indicate that BMP2b is required not only for the initial migration of NCCs from the vagal crest to the anterior end of the intestine, but also for ENS development along the intestine. Additionally, we found that BMP2b is required for the development of smooth muscles in zebrafish. With respect to the molecular mechanism involved, we demonstrate that BMP2b is required for the normal proliferation of vagal NCCs as a mitogen that gives rise to the ENS precursors, a role that is mediated by GDNF in the intestine.

## Materials and Methods

### Ethics Statement

The study was approved by the Ethical Committee Psychology (ECP) affiliated with the Chongqing Medical University, China (for this study, the approval number is 2016-059). All guardians of the participants gave written informed consent before the study. All guardians of the participants had the right to refuse or stop participating during the study. The remaining experiments were performed on rats and zebrafish and were approved by the animal care and use committee of Chongqing Medical University. All of the experiments were performed in accordance with the government policies and the approved guidelines.

For the animal experiment, SD rats (SPF grade) were fed in the animal center of Chongqing Medical University, where the animal work was performed. This housing facility is an ordinary housing facility, and it is in keeping with the national standard “Laboratory Animal-Requirements of Environment and Housing Facilities.” The care of laboratory animals and the animal experimental operations conformed to “Chongqing Administration Rule of Laboratory Animal.”

### Clinical Cases for HSCR

The pathological diagnosis of HSCR was confirmed by hematein–eosin staining and Calretinin immunohistochemistry (Guinard-Samuel et al., 2009). This case series consisted of 40 HSCR patients and 10 controls. All HSCR cases attained from 31 (77.5%) males and 9 females were affected with short-segment disease. The age ranged from 1 month to 6 years (mean 10.2 months). 37.5% (15/40) of the cases were younger than 3 months old, 52.5% (21/40) of the cases were 3 months to 1 year old, 10.0% (4/40) of the cases were more than 1 year old at presentation. Another 10 cases were control (seven samples from children who died of non-digestive diseases, three samples from colostomy due to rectal trauma) intestinal samples, mature ganglion cells found in the myenteric and submucosal plexus with H & E staining. Control subjects were sex- (73.3% males) and age-matched normal children. Both cases and controls were Chinese Han in Chongqing, and informed consent was obtained.

### Preparing NCCs Medium

To culture NCCs from E11.5 SD rat guts (approximately one litter, depending on the genetic background of the mouse line), prepare 25 mL of NCCs medium. Combine 12.5 mL low glucose DMEM/F12 (Sigma, DF042), 7.5 mL Neurobasal Medium (Sigma, B1522), 25 μL retinoic acid (117 μM final concentration, Sigma, R2625), and 25 μL 2-mercaptoethanol (50 mM final concentration, Sigma, 07604), mix well. Add 3.75 mL Chick Embryo Extract (Accurate Chemical), 250 μL N2 salt supplement (Gibco, 17502001), 500 μL B27 supplement (Gibco, 17504044) and 250 μL penicillin-streptomycin (1% final concentration). Filter the medium through a 0.22 μm filter. Add 10 μL sterile recombinant human IGF1 (20 μg/mL final concentration, Gibco, PHG0078) and 20 μL sterile recombinant murine bFGF (20 μg/mL final concentration, Gibco, 13256029), mix by inverting. Store at 4°C.

### Neurosphere Culture

E11.5 SD rat guts were dissected in DMEM/F12 medium, washed with PBS and digested with type I collagenase (750 U/Ml, Sigma, 1148089) and dispase (250 ug/mL, Roche, 04942086001). Then the digested guts were triturated and filtered through cell strainers, washed with DMEM/F12, resuspended in NCCs medium, and plated on poly-D-lysine- and fibronectin-coated wells. The culture medium was changed every 3 days. Cells were cultured in a humidified incubator at 37°C and 5% CO_2_. For cell differentiation, neurospheres were seeded on 48-well cell culture plates coated with 5 mg/cm2 rat tail collagen type I (BP Bioscience), covered with glass cover slips, and cultured for up to 3 days in NCCs medium and 10% fetal calf serum. For the NCCs proliferation experiment, the concentration of BMP2 was from 10 to 120 ng/mL. To identify the role of BMP2 and GDNF in the differentiation of NCCs, BMP2 (50 ng/mL, Abcam, ab87065) and GDNF (100 ng/mL, Abcam, ab73450) were added separately or in combination in different groups.

### Collagen Gel Culture

As in the previous study, three-dimensional collagen gel culture was performed following the procedure of Fu et al. (2006). Briefly, 1 vol of acid-soluble collagen solution (4 mg of typeIcollagen gel dissolved in 1 ml of 0.2% acetic acid) was mixed with 4 vol of DMEM/F12/10% FCS (Sigma, F8192). Then, 0.8 M NaHCO_3_ (Sigma, S5761) and 5 × DMEM/F12 200 mM NaOH (Sigma, 655104) was added to the solution to bring the pH to approximately 7.8. Finally, the solution contained collagen (750 μg/mL), FCS (7.5%), NaHCO3 (6 mM), and DMEM/F12 (1×). For this experiment, either BMP2 (50 ng/mL), GDNF(100 ng/mL), or both were added directly to the gel. Then, 300- to 400-μm midgut explants from E11.5 mouse embryos were placed into the collagen gel and cultured in a humidified incubator at 37°C and 5% CO_2_ for 48 h, before fixation with 4% paraformaldehyde (PFA).

### Zebrafish Maintenance and Breeding

Fish were maintained and used under standard conditions at 28.5°C (Kimmel et al., 1995). To better observe the internal structures of the embryos, the embryos were incubated with 0.2 mM 1-phenyl-2-thiourea (Sigma) from 24 hpf to block pigment development. The transgenic zebrafish line, Tg(hsp70:BMP2b-GFP) was a gift from Didier Y. R. Stainier, and Tg(Foxd3: GFP) was obtained from Georg-August-University of Gottingen.

### Embryonic Microinjections

The BMP2b MO antisense oligonucleotide (Gene Tools) was designed to target the splicing of BMP2b mRNA based on the following sequence: 5′-CAAATCAGGCCTCACCTTCGTGATG-3′ (Wise and Stock, 2010). The GDNF MO antisense oligonucleotide (Gene Tools) was designed to target the splicing of GDNF mRNA based on the following sequence: 5′-TGTCCCATAACTTCATTTTAGACT-3′ (Shepherd et al., 2001). The standard control MO (cont MO: 5′-CCTCTTACCTCAGTTACAATTTATA-3′) was used as control for MO injection. The BMP2b MO, GDNF MO, and Cont MO were separately diluted to working concentration (GDNF MO, 200 μM; BMP2b MO, 300 μM; Cont MO, 300 μM). Approximately 1 nl diluted MO was injected into the embryos (1-4 cell stage). To synthesize GDNF mRNA and BMP2b mRNA *in vitro*, we cloned the coding sequence (CDs) of zebrafish GDNF/BMP2b into the vector pCS2 +, sequencing confirmed the correct clones were prepared. Then the plasmids were linearized by *Not*I digestion individually and the capped GDNF mRNA/BMP2b mRNA was synthesized according to the manual [mMESSAGE mMACHINE Kit (AM1340, Ambion)]. GDNF mRNA(40 ng/μl)/BMP2b mRNA(30 ng/μl) was injected at the 1–4 cell stage, and approximately 1 nl diluted mRNA was injected into each embryo.

### BrdU Staining

BrdU staining (Takagi et al., 1993) was performed in order to detect the NCCs proliferation in different groups. 10 mM BrdU (Abcam, ab142567) stock solution was diluted in a cell culture medium to make a 10 μM BrdU labeling solution. The 10 μM BrdU labeling solution was filtered through a 0.2 μm filter under sterile conditions. The existing culture medium was removed from the cells and replaced with 10 μM labeling solution. The cells in the BrdU labeling solution were incubated for 2 h at 37°C in a CO_2_ incubator. BrdU labeling solution was removed from the cells and were then washed twice in PBS. Cells were fixed in 4% PFA at room temperature and washed twice in PBS. Cells were incubated with 1% BSA in PBS at room temperature for 30 min. Then, cells were incubated with a 1:500 dilution of rabbit polyclonal anti-BrdU primary antibody (Abcam, ab152095) at 37°C for 2 h. Cells were washed in PBS twice and then incubated with a 1:1000 dilution of Alexa Fluor 647 anti-rabbit IgG antibody (Abcam, ab150075) at 37°C for 1 h. Cells were washed in PBS for twice and mounted with 50% glycerol (Sigma, G5516).

### Immunohistochemistry

Immunohistochemistry was performed as previously described (Raible and Kruse, 2000). In brief, paraffin sections were first rehydrated, and then rehydrated sections were incubated with a 1:500 dilution of rabbit anti-human primary antibody against BMP2 (1:300, Abcam, ab14933) and GDNF (1:200, Abcam, ab18956), or IgG (1:1000, as a negative control, Abcam, ab172730) overnight at 4°C. The tissue sections were washed in PBS and then incubated with a 1:100 dilution of biotinylated secondary goat anti-rabbit IgG (1:1000, Jingmei BioTech, Shenzhen, China). After washing with PBS, tissue sections were incubated with an avidin-biotin complex and developed in 0.075% (w:v) 3,3 diaminobenzidine (DAB). After lightly counterstaining with hematoxylin, the sections were dehydrated.

### Immunofluorescence

Immunofluorescence was performed using a mouse monoclonal anti-GFP antibody (1:500, Abcam, ab38689) and a rabbit polyclonal anti-GFP antibody (1:500, Abcam, ab6556). NCCs were identified using rabbit monoclonal anti-p75 NGR antibody (1:50, Abcam, ab52987), rabbit monoclonal anti-Nestin antibody (1:100, Abcam, ab105389), mouse monoclonal anti-beta III Tubulin (Tuj1) antibody (1:300, Abcam, ab109016), rabbit monoclonal anti-GFAP antibody (1:300, Abcam, ab33922), rabbit polyclonal anti-α-SMA antibody (1:100, Abcam, ab5694), and rabbit monoclonal anti-Hu antibody (1:500, Abcam, ab232416). Proliferating cells were identified using a monoclonal anti-phosphohistone H3 antibody (1:500, Abcam, ab1791). Cells undergoing apoptosis were identified using an anti-activated Caspase-3 rabbit polyclonal antibody (1:200, Abcam, ab13847). Rabbit antibodies were visualized using an Alexa Fluor 647 or an Alexa Fluor 488 anti-rabbit IgG antibody (1:500, Abcam, ab150077) and the mouse anti-GFP antibody was visualized using an Alexa Flour 488 anti-mouse IgG antibody (1:500, Abcam, ab150125). Immunofluorescence samples were processed following a previously described protocol (Mccaffery and Farquhar, 1995). Briefly, cultured cells were fixed for 15 min at room temperature in 4% PFA, and then washed in PBS twice. Cells were permeated at room temperature for 15 min with 0.5% Triton X-100 (Sigma, T8787). Absorbent paper was blotted with PBS, 5% normal serum was added, and the paper was incubated at room temperature for 1 h. After removing the blocking solution, sufficient and diluted primary antibodies were added and samples were placed in a wet box and incubated overnight at 4°C. The samples were washed in PBS twice and then incubated with the diluted fluorophore-conjugated secondary antibodies at 37°C for 1 h. The samples were then washed in PBS twice and mounted with antifade mountant. Observation and images collection was performed using a fluorescence microscope.

### Flow Cytometer

Primary NCCs were collected and washed with PBS 3 times. Unlabeled cells were divided into four groups and treated with or without mouse monoclonal to P75^*NGF*^ (PE/Cy7) antibody (1:100, Abcam, ab234270), mouse monoclonal to CD49d (FITC) antibody (1:150, Abcam, ab221), or with both at room temperature for 25 min. Cells were then washed with PBS 3 times, pelleted and resuspended in 1.0 ml PBS. A FACSCanto II FCM (Becton Dickinson, Broendby, Denmark) was immediately used to collect data for all groups, and analysis was performed using FACSDiva software 5.3 (Becton Dickinson) to determine the intensity and percentage of DCF fluorescent cells (FL1-H) among living cells.

### Whole-Mount *in situ* Hybridization

Embryos were prepared for whole-mount ISH as previously described (Thisse et al., 1994). Most of the plasmids were from Luo lab. Digoxigenin-labeled riboprobes were synthesized from a linearized plasmid as follows: crestin (Rubinstein et al., 2000); phox2b (Elworthy et al., 2005); α-SMA (Georgijevic et al., 2007); GDNF (Shepherd et al., 2001). The probes for identifying zebrafish *BMPRIa* and *BMPRIb* were amplified from cDNA pools of 72hpf embryos using the appropriate sets of primers. The sequence of the primers is outlined in Supplementary Table S1. Then the amplified PCR products were ligated with pGEM T-easy vector, the successful plasmids were linearized and the probes were synthesized according to the manual (Roche,11487671001). Before use in ISH experiments, embryos were fixed overnight at 4°C in 4% PFA in PBS, washed with MetOH(100%) four times and stored in 100% MetOH at −20°C. The most important step of our ISH method was as follows: embryos were hybridized with the Dig-labeled RNA probe in a hybridization buffer (50% formamide, 5XSSC, 50 μg/ml tRNA and 0.1% Tween 20) at 67°C, followed by incubation with anti-Dig antibody conjugated with alkaline phosphatase (AP) and staining with the substrates NBT and BCIP.

### Real-Time PCR

Total RNA was extracted and purified from the samples, and cDNA was synthesized according to the manual (Takara,6210B,PrimeScript^TM^ II 1st Strand cDNA Synthesis Kit). For real-time PCR, amplifications were detected using Fast SYBR Green Master mix in reactions performed with the iCycle iQ PCR Detection system (Bio-Rad). Each 20 μl PCR reaction contained 1 × Fast SYBR Green Master mix, 0.2 μM of each primer pair, and 2 μl of each first-strand cDNA template. PCR reactions were started with an initial denaturation at 94°C for 3 min, followed by 35 cycles of denaturation at 94°C for 30 s, annealing at 57°C for 30 s, and extension at 72°C for 30 s. Melting curve analysis was performed after amplification was completed. The mRNA amount of each target gene was then normalized to the respective control β-actin amounts and presented as a ratio under different conditions. The sequence of the primers is outlined in Supplementary Table S2.

### Western Blotting

Approximately 50 mg samples were minced to small pieces using surgical blades and sonicated in a protein lysis buffer containing protease inhibitors (complete, Ultra, Mini, EDTA-free, EASYpack Roche, Germany). Protein concentrations were measured by the BCA method, and samples were adjusted to the same protein concentration, aliquoted and stored at −80°C. Equal amounts of total proteins from tissues were separated on SDS-polyacrylamide gels and electro-transferred to PVDF membranes (Roche Germany). The blots were incubated with following polyclonal antibodies overnight at 4°C: anti-BMP2 (1:1000, Abcam), anti-GDNF (1:1000, Abcam), and anti-pSmad1/5/8 (1:1500, Abcam). Blots were washed and incubated with horseradish peroxidase-linked secondary antibodies (1:2000, Abcam) for 2 h at room temperature and detected using ECL reagent (Millpore, MA, United States).

### Heat-Shock Experiments

To test the role of BMP signal in the development of ENS precursors in zebrafish, transgenic embryos [Tg (hsp70: BMP2b-GFP)] (Shin et al., 2007) at 10 hpf were incubated at 39°C for 40 min, then were transferred into the medium at 28.5°C and cultured to 72 hpf. GFP-positive and GFP-negative controls were collected and fixed for phox2b expression studies. For rescue experiments, Tg (hsp70: BMP2b-GFP) heterozygous embryos were injected with BMP2b MO and heat-shocked at 10 hpf. Fertilized eggs were injected with BMP2b MO without heat-shock as the control group. All of the embryos were cultured to 72 hpf and fixed overnight. Then, phox2b expression was assessed by ISH.

### Statistics

Results are expressed as the mean ± SE for each group. A significance value of *P* < 0.05 was specified to be significant. A two-tailed Student’s *t*-test was used for pair-wise comparison. Two-way ANOVA was used for comparisons among multiple groups. Analysis was performed using SAS 9.0 or GraphPad Prism 8. The statistics were checked by a biostatistics expert.

## Results

### BMP2 Promotes NCC Proliferation, Migration, and Differentiation

#### Expression of BMP2 and GDNF in HSCR

**FIGURE 1:**
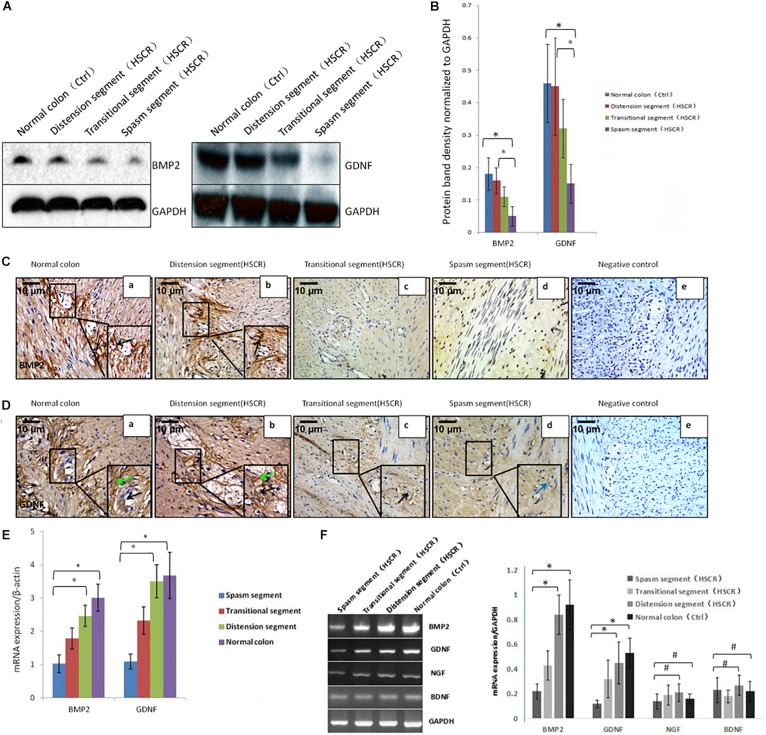
Low or no expression of BMP2 and GDNF in the spasm segment of Hirschsprung disease. **(A)** Western blot analysis of BMP2 and GDNF expression in normal colon and different segments of HSCR. **(B)** The density of BMP2/GDNF in different HSCR segments and normal colon. The protein expression of BMP2 in the spasm segment of HSCR is approximately 1/3 fold the density of distension segment of HSCR and normal colon (*n* = 3, *t*-test, ^∗^*P* < 0.05, compared with normal colon and distension segment of HSCR); The protein expression of GDNF in the spasm segment of HSCR is approximately 1/4 fold the density of distension segment of HSCR and normal colon (*n* = 3, *t*-test, ^∗^*P* < 0.05, compared with normal colon and distension segment of HSCR). **(C)** Immunohistochemistry was done to detect the expression of BMP2 in normal colon and different segments of HSCR. BMP2 was highly expressed in myenteric nerve plexuses of the normal colon **(a)** and distension segment of HSCR **(b)**, but minimally expressed in the transitional segment **(c)**, and spasm segment of HSCR **(d)** (black arrow: positive expression of BMP2 in ganglion cells). **(D)** Immunohistochemistry was done to detect the expression of GDNF in normal colon and different segment of HSCR. GDNF was strongly expressed in the endoderm but minimally expressed or absent in the nerve plexus **(a,b)** and the ectoderm of spasm segment **(d)**. **(E)** Real-time PCR analysis of BMP2 and GDNF expression in normal colon and different segments of HSCR. The mRNA expression of BMP2 in the spasm segment of HSCR is approximately 1/3 fold of distension segment of HSCR and normal colon (*n* = 3, *t*-test, ^∗^*P* < 0.05, compared with normal colon and distension segment of HSCR); The mRNA expression of GDNF in the spasm segment of HSCR is approximately 1/4 fold of distension segment of HSCR and normal colon (*n* = 3, *t*-test, ^∗^*P* < 0.05, compared with normal colon and distension segment of HSCR). **(F)** PCR analysis of BMP2, GDNF, NGF, and BDNF expression in normal colon and different segments of HSCR. The expression of BMP2 and GDNF was decreased in the spasm segment of HSCR. The mRNA expression of BMP2 or GDNF in the spasm segment of HSCR is approximately 1/4 or 1/5 fold the density of distension segment of HSCR and normal colon (*n* = 3, *t*-test, ^∗^*P* < 0.05, compared with normal colon and distension segment of HSCR); There is no significant difference in the density of mRNA expression in NGF and BDNF between the spasm segment of HSCR and the distension segment of HSCR and normal colon (*n* = 3, *t*-test, ^#^*P* > 0.05, compared with normal colon and distension segment of HSCR); (black arrow: low or no expression of GDNF in ganglion cells; green arrow: positive expression of GDNF in the endoderm of normal colon and distension segment of HSCR; blue arrow: low expression of GDNF in the endoderm of spasm segment of HSCR).

To study the difference in the activity of core signaling between patient tissue samples and controls, we used the PCR method to assess the transcription of BMP2, GDNF, NGF, and BDNF. BMP2 and GDNF expression was reduced in the spasm segment, whereas NGF and BDNF expression was not altered (Figure 1F). Western blot and real-time PCR were further performed to assess BMP2 and GDNF expression. The results showed that BMP2 and GDNF expression was significantly decreased in the spasm segment compared with the normal colon and the distension segment (Figures 1A,B,E). To confirm the downregulated activity of BMP signaling in tissue samples from cases, we further assessed p-Smad1, p-Smad5, and p-Smad8 levels. No significant differences in p-Smad5 and p-Smad8 expression were noted, but p-Smad1 levels were reduced in the cases compared with the controls (Supplementary Figure S1).

BMP2 was expressed in the normal colon (Figure 1C,a) and the distension segment (Figure 1C,b), but was minimally expressed in the transitional segment (Figure 1C,c) and the spasm segment (Figure 1C,d). GDNF was strongly expressed in the normal colon (Figure 1D,a) and the distension segment (Figure 1D,b), but minimally expressed in the transitional segment (Figure 1D,c) and spasm segment (Figure 1D,d). Our results demonstrated that BMP2 and GDNF are typically expressed in the ENS, and their reduced expression in the gut of aganglionosis patients suggested that these proteins participate in the development and maturation of the ENS.

#### Identification of NCCs

**FIGURE 2:**
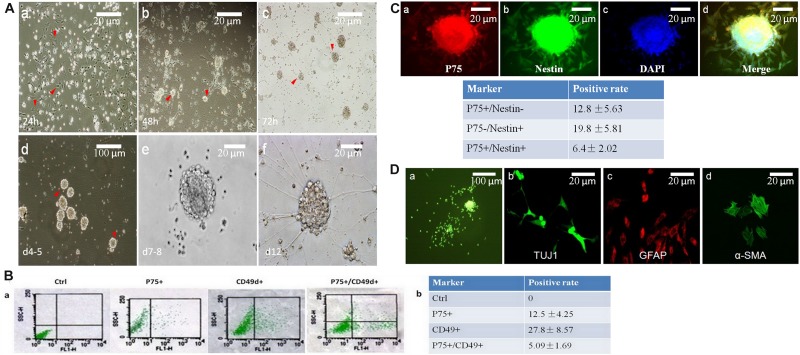
Neurospheres maintain the charactors for self-renewable and multipotent NCCs. **(A)** Primary neurospheres were formed at day 7-8**(A,e)**. At 24 h, cells grew with adherence (**A,a**, red arrow head). After replating, tiny clusters can been seen at day 2 (**A,b**, red arrow head), which then became a dome (**A,c**, red arrow head) and neurospheres on day five (**A,d**, red arrow head). **(B)** Flow cytometry was used to detect the percentage of P75+ or CD49d+. The positive rate of P75+/CD49d+ was 5.09%. **(C)** The percentage of P75+/Nestin+ cells were 6.4%, which was detected by immunofluorescence. **(D)** Primary neurospheres were differentiated into neuron (TUJ1+), glial cells (GFAP+), and smooth muscle cells (α-SMA+).

Neural crest cells were isolated from E11.5 mouse guts. The cells were cultivated under serum-free conditions and the culture medium was supplemented with IGF1 and bFGF. This approach resulted in the formation of neurospheres. Initially, both NCCs and non-NCCs (mainly mesenchymal cells) grew as adherent cells (Figure 2A,a). Due to the serum-free conditions, mesenchymal cells exhibited poor growth, and these cells were removed at each replating (Figure 2A,b). Clusters of cells were first observed on day 3 (Figure 2A,c). These clusters grew faster and formed primary neurospheres on days 4-5 (Figure 2A,d). The neurospheres were digested into single cells, and these clusters grew into secondary neurospheres on days 7–8 (Figure 2A,e).

P75 is a low-affinity NGF receptor, that is mainly expressed in the PNS. CD49d is a transmembrane protein that mediates interactions between adhesion molecules on adjacent cells and/or the extracellular matrix. CD49d plays diverse roles in cell migration during development and cell differentiation. Nestin is a class VI intermediate filament protein that is expressed in dividing cells during the early stages of development in the CNS, PNS and myogenic cells. So, in this study, P75^+^/CD49d^+^ and P75^+^/Nestin^+^ double-stained cells were regarded as NCCs. To identify the positive rates of NCCs, flow cytometry was used to detect the percentages of P75- and CD49d-positive cells (12.5 and 27.8%, respectively; Figures 2B,a,b). The percentage of P75^+^/CD49d^+^ double-stained cells was 5.09% (Figures 2Ba,b), indicating that the percentage of NCCs was 5.09%. In addition, immunofluorescence data revealed that the percentage of P75^+^/Nestin^+^ double-stained cells was 6.4% (Figure 2C).

To investigate the multi-directional differentiation of NCCs, immunofluorescence analysis was performed, and the results demonstrated that primary neurospheres were differentiated into the neuron (TUJ1^+^), glial cells (GFAP^+^) and smooth muscle cells (SMA +) (Figure 2D).

In conclusion, these data indicated that NCCs were successfully obtained *in vitro*.

#### BMP2 Promotes NCC Proliferation, Migration, and Differentiation

**FIGURE 3:**
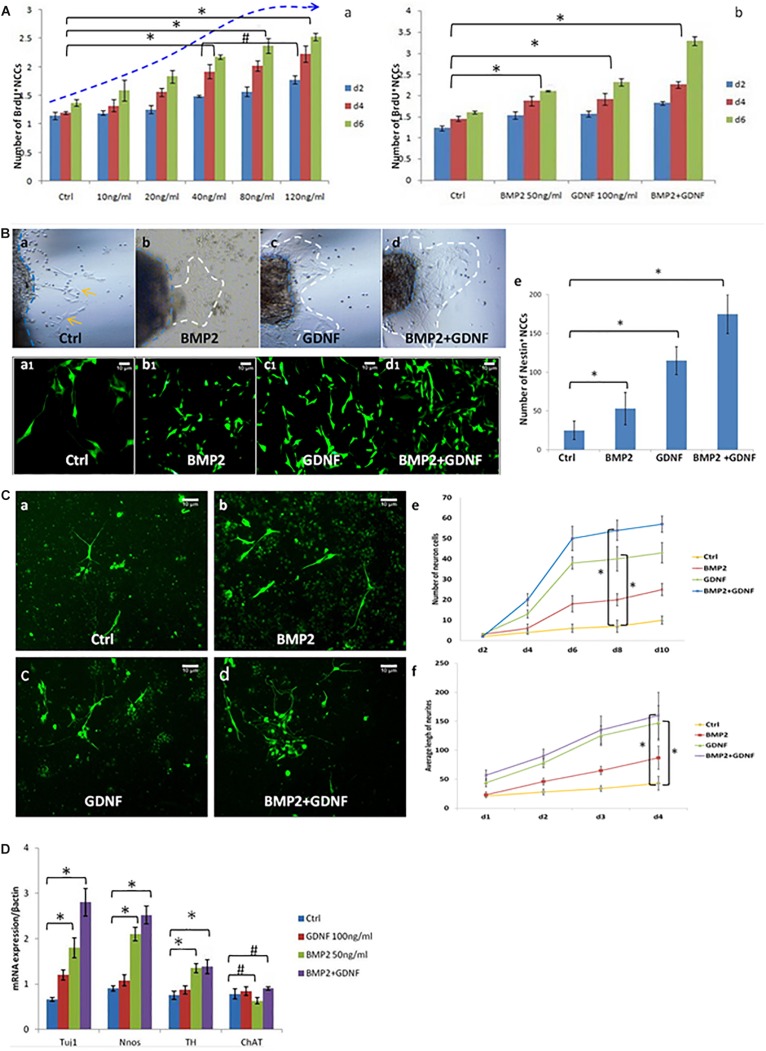
BMP2 promotes the proliferation migration and differentiation of NCCs. **(A)** BMP2 promote NCCs proliferation in a concentration-dependent manner. **(A,a)** Exposure to concentrations of BMP2 between 10 and 80 ng/ml for 2, 4, and 6 days significantly promoted NCC proliferation. There was no significant difference of NCC proliferation in the BMP2 concentration between 80 and 120 ng/ml (*n* = 3, 2-way ANOVA, ^∗^*P* < 0.05, compared with ctrl; ^#^*P* > 0.05, compared with 40 ng/ml group); **(A,b)** BMP2 and GDNF significantly promoted NCC proliferation (*n* = 3, 2-way ANOVA, ^∗^*P* < 0.05, compared with ctrl). **(B)** Both BMP2 and GDNF promote migration of Nestin^+^ NCCs. Cells were treated with DMSO, GDNF, BMP2, or both GDNF and BMP2 for 2 days. Compared with control group **(B,a,a1)**, there were more Nestin + NCCs migrating into the collagen gel **(B,b,b1,c,c1,d,d1)**; **(B,e)** Values are given as mean ± SE. *n* = 3, *t*-test, ^∗^*P* < 0.05, compared with control. **(C)** Immunofluorescence images showing primary enteric neurons (Tuj1^+^) cultured when treated with GDNF, BMP2, or both. Cells were treated with DMSO, GDNF, BMP2, or both GDNF and BMP2 for 2 days. Primary enteric neurons were labeled with Tuj1. **(C,e)** The number of neuron cells were counted. Values are given as mean ± SE. *n* = 3, 2-way ANOVA, ^∗^*P* < 0.05, compared with ctrl. **(C,f)** The average length of neuritis were measured. Values are given as mean ± SE. *n* = 3, 2-way ANOVA, ^∗^*P* < 0.05, compared with ctrl. **(D)** Real time-PCR was done by using RNA from NCCs treated with GDNF, BMP2, or both GDNF and BMP2 for 48 h and probed for different genes. A clear increase in TUJ1 expression was noted in cells cultured in the presence of BMP2 or GDNF. BMP2 also promoted the expression of nNOS and catecholaminergic neurons (TH expression) but had no effect on the expression of ChAT. Values are given as mean ± SE, *n* = 3, *t*-test, ^∗^*P* < 0.05, compared with BMP2 and BMP2 + GDNF treatment group; ^#^*P* > 0.05, compared with BMP2 and BMP2 + GDNF treatment group.

To study the role of BMP2 in regulating the growth and differentiation of NCCs, we cultured neurospheres with and without BMP2. Exposure to concentrations of BMP2 between 10 and 80 ng/ml for 2, 4 and 6 days significantly promoted NCC proliferation in a concentration-dependent manner (Figure 3A,a). Additionally, BMP2 and GDNF significantly promoted NCC proliferation compared with the control (Figure 3A,b and Supplementary Figure S2). Together BMP2 and GDNF enhanced NCC proliferation.

To study the effect of BMP2 on NCC migration, we established a three-dimensional culture of the intestinal segment (E11.5). In the presence of BMP2 or GDNF, more NCCs (Nestin was detected as a marker by immunofluorescence) migrated into the collagen gel (Figures 3B,b,c,b1,c1,e). The addition of BMP2 and GDNF further enhanced NCC migration (Figures 3B,d,d1,e). Our results suggested that both BMP2 and GDNF promote NCC migration.

We continued to study the role of BMP2 and GDNF in the differentiation of primary NCCs by observing neurite length and neurite bearing cell numbers. Cells were treated with either DMSO, GDNF, BMP2, or both GDNF and BMP2, for 2 days. Primary enteric neurons were labeled with Tuj1. The results showed that BMP2 and GDNF treatment promoted enteric neuronal differentiation compared with control (Figure 3C), and co-treatment with BMP2 and GDNF enhanced NCC differentiation (Figure 3C).

We next assessed the effect of GDNF and BMP2 on the expression of different neuronal markers using quantitative real-time PCR. A clear increase in TUJ1 expression was noted in cells cultured in the presence of BMP2 or GDNF compared with the control group (Figure 3D). BMP2 also promoted the expression of nNOS and catecholaminergic neurons (TH expression) but had no effect on the expression of ChAT, a cholinergic neuron-specific marker.

In conclusion, the above data demonstrated that BMP2 promotes NCC proliferation, migration and differentiation.

### BMP2 Is Required for ENS Development in Zebrafish

#### Migration of ENS Precursors Through the Digestive System

The *in vitro* experiments described above indicated that BMP2 is involved in the development of NCCs. Hence, we used zebrafish as a model to further study the role of BMP2 in the development of the ENS.

Phox2b is expressed in differentiating neurons and their precursors (Dijke et al., 2002; Elworthy et al., 2005) and has been used to assess enteric precursor migration. Crestin has been used to identify early enteric precursors (Luo et al., 2001). In this study, we first observed the migration of ENS precursors by studying the expression of phox2b and crestin. The data showed that crestin was first expressed in the majority of enteric precursors at 34 h post-fertilization (hpf) (Supplementary Figure S3A,a). Phox2b was expressed in the two lateral lines of enteric precursors, which is consistent with previous studies (Shepherd et al., 2004; Goldstein et al., 2005). Enteric precursors migrated to a portion of the anterior intestine by 52 hpf (Supplementary Figure S3B,a). As the embryos developed, enteric precursors migrated farther posterior (Supplementary Figures S3B,b,c) and reach the end of the intestine by 66 hpf (Supplementary Figure S3B,d).

#### Expression of BMP2 and BMP2 Signaling Pathway Components During ENS Development

To determine whether BMP2 and components of its receptor, BMPR1a and BMPR1b, exhibit expression patterns that correlate with the migration of ENS precursors from the vagal crest region to the anterior end of the intestine, we examined the expression patterns of these genes. BMP2b, which plays a key role during development, is first expressed at the anterior end of the intestine around 34 hpf (Supplementary Figures S4A,B). At 60 hpf, BMP2b expression was observed along the intestine from the anterior region to the posterior end(Supplementary Figures S4E,F). BMPR1a was diffusely expressed throughout the endoderm adjacent to the mesenchyme and was expressed by ENS NCCs (Supplementary Figures S4C,D,G,H,K,L). BMPR1b was also expressed throughout the mesenchyme (Supplementary Figures S4I,J). These patterns of BMP2b and BMPR1a/1b expression are consistent with BMP2b playing a direct role in ENS development in the zebrafish.

#### Requirements for BMP2b Expression in Zebrafish ENS Development

**FIGURE 4:**
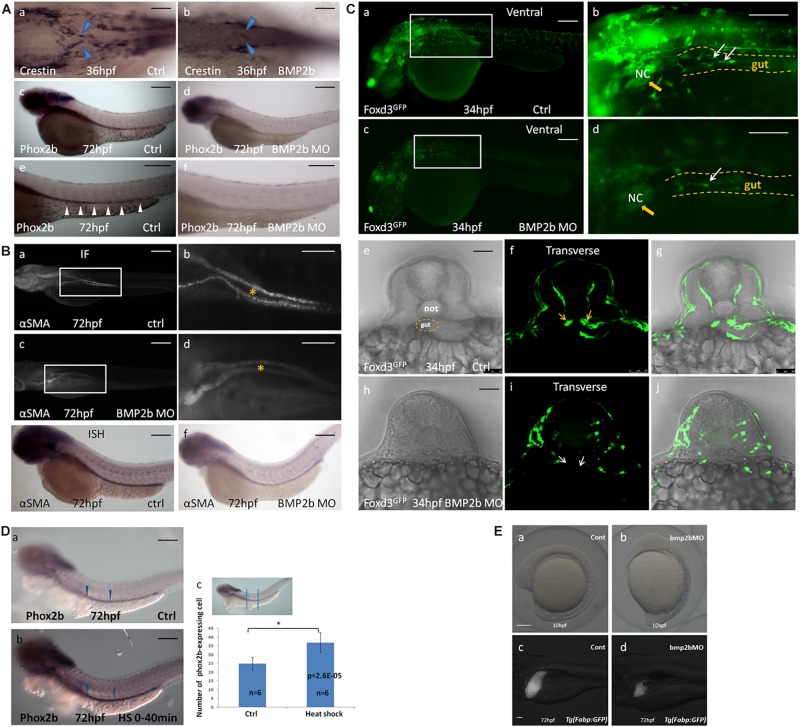
BMP2b is required for normal ENS and intestinal smooth muscle development. **(A,a,c,e;B,a,b,e;C,a,b,e,f,g)** wild-type embryos, **(A,b,d,f;B,c,d,f;C,c,d,h,i,j)** BMP2b MO embryos. **(A,b)** Dorsal viewing for the vagal region of 36 hpf embryos hybridized with crestin probe showed a defective vagal NCC migration to the anterior end of the intestine in BMP2MO morphants(26/37, 70.3%). **(A,d,f)** Lateral view of 60 hpf embryos hybridized with phox2b probe showing a failure of precursors migration to the anterior of the intestine in BMP2MO morphants(26/34, 76.5%). **(B,c,d)** Immunofluorescence showed the developmental defect of intestinal smooth muscle in the intestine of BMP2b MO embryos(22/30, 73.3%). **(B,f)** Lateral view of the intestine for 72 hpf embryos hybridized with α-SMA showing a reduce of α-SMA expressing in the intestine of BMP2b MO embryos(31/39, 79.5%). **(C,c,d,h,i,j)** Lateral and transverse view the vagal region of 34 hpf embryos labeled with GFP for foxd3 showed a failure of vagal NCC migration in BMP2MO morphants(29/36, 80.5%). **(D)** BMP2 overexpression results in an increase in enteric neurons. Lateral views of phox2b in enteric ganglia of 72 hpf control **(D,a)** and HS embryos **(D,b)**. **(D,c)** Bar graph summarizes the results from one of three independent BMP2 HS experiments (six zebrafish in different groups). The phox2b expressing cells in the HS group is approximately 1.5 fold of ctrl group (*n* = 6, *t*-test, ^∗^*P* < 0.05, compared with ctrl group). **(E)** BMP2b is necessary to maintain the anterior-posterior axis of embryos display and liver development. **(a)** control embryos(114/120, 95%). **(b)** BMP 2b MO treated embryos that have the anterior–posterior axis of embryos displayed defect(43/60, 71.7%). **(c)** control embryos(68/80, 85%). **(d)** BMP2b MO treated embryos, the liver became smaller on day 3(51/76, 67.1%). Bar, 100 μm. Notice: here “26/37” means 26 out of the 37 embryos had defective NCC migration like in the image showed. The following statistical numbers for embryos like this have the same meaning in the Figure legends. Blue arrows **(A)** indicate the migrating enteric precursors. White arrowheads **(A)** indicate phox2b expressing cells in the intestine. Asterisk **(B)** indicate gut. Yellow arrowheads **(C)** indicate vagal region. White arrows **(C)** indicate the migrating enteric precursors.

To identify whether BMP2b is required earlier in ENS development, we synthesized a BMP2b antisense MO and a standard control MO. We injected the BMP2b MO and control MO at the 1- to 4 cell stage then collected embryos at 24 hpf. Western blotting was performed to assess whether the BMP2b MO was effective. The data showed that the newly synthesized BMP2b MO worked well (Supplementary Figure S5). In addition, when BMP2b was knocked down, the anterior-posterior axis of the embryos displayed a defect (Figures 4E,a,b), and liver size was reduced on day 3 (Figures 4E,c,d), which was similar to data published in earlier reports (Ramel et al., 2005; Chung et al., 2008), further confirming the efficiency of the Bmp2b MO. Then, we performed hybridization experiments in wild-type and BMP2b MO zebrafish embryos at 34 hpf using a probe for crestin (Figure 4A,a) and at 60 hpf using a probe for phox2b (Figures 4A,c,e). No distinct migrating ENS precursors were observed in the MO embryos (Figures 4A,a,d,f). To further identify whether other aspects of intestinal development are disordered in MO embryos, we studied intestinal muscle layer development in these embryos. Compared with wild-type embryos (Figures 4B,a,e), the expression of the smooth muscle marker αSMA was severely disturbed in BMP2b MO embryos (Figures 4B,d,f).

Foxd3 exhibits highly conserved neural crest expression before neural crest migration throughout vertebrates (Sasai et al., 2001), and helps to maintain the pluripotency of the neural crest (Montero-Balaguer et al., 2006). Thus, as a conserved marker, foxd3 can be used to indicate neural crest migration. In this study, the *Tg (foxd3: gfp)* transgenic zebrafish line was used to determine the function of the BMP2b signal in neural crest migration. At 34 hpf, a large mass of enteric precursors (labeled by GFP) migrated from the vagal region to the anterior region of the gut (Figures 4C,a,b,f). However, in the BMP2b MO group, the vagal region was poorly developed. A few cells migrated to the anterior of the gut, and the precursors exhibited an abnormal morphology (Figures 4C,c,d,i). This data indicated that losing the function of BMP2b reduced the early neural crest migration and the overall migration of NCCs.

Given that the loss of BMP2 signaling results in a reduction in ENS precursors, we wanted to assess whether overexpression of BMP2 in early embryogenesis increases the number of ENS precursors. The Hsp70: BMP2b-GFP transgenic zebrafish line was used to identify whether BMP2b affects ENS precursors *in vivo*. At 10 hpf, zebrafish were subjected to heat shock (40 min at 39°C) to activate BMP2b. Then, the number of ENS precursors in the embryos was detected on the basis of phox2b expression at 72 hpf. The results indicated that the extent of phox2b expression along the anterior-posterior axis of the gut did not differ significantly between the control embryos and the heat-shocked embryos, while the intensity of phox2b staining in the phox2b-positive precursors in the intestine increased, with an intensity of 47 ± 4.5 being observed for the phox2b-expressing ENS precursors among heat-shocked embryos vs. 28 ± 3.7 among the controls in the intestine (Figures 4D,a,b,c). This result further confirmed that BMP2b was involved in ENS development.

#### Proliferation of Vagal ENS Precursors Is Reduced in BMP2b MO Embryos

**FIGURE 5:**
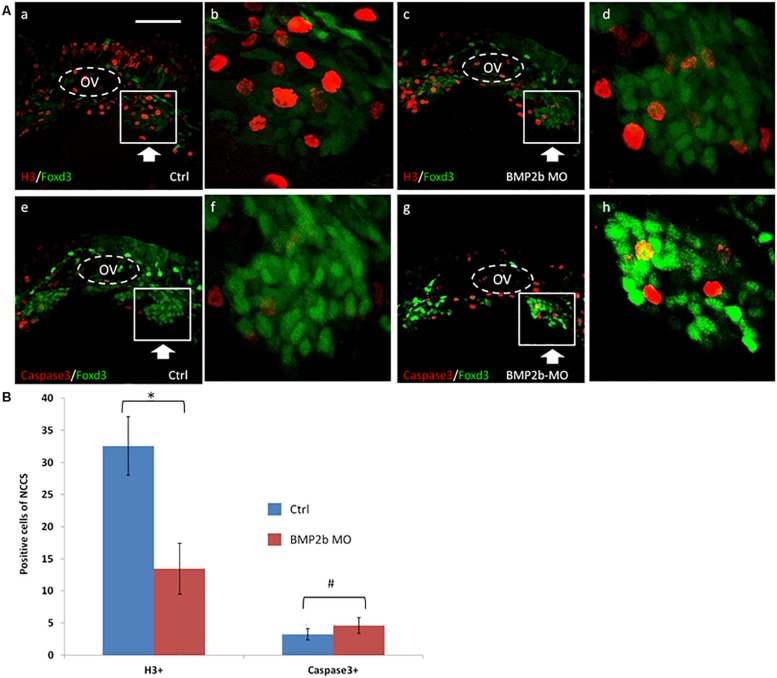
Proliferation but not apoptosis was changed in vagal NCC and ENS NCC in BMP2b morphants. Here we selected the areas included in the squares to count the positive cells, and the square areas were magnified on the right side of each image. **(A,b,f)** Lateral views of the vagal region of 30 hpf wild type(11/11, 100%)and **(A,d,h)** BMP2b MO treated (29/33, 87.9%)Foxd3:GFP transgenic embryos. Embryos were double stained with anti gfp (green) to study the distribution of vagal NCC and ENS NCC’s, anti caspase 3 (red) **(A,e,h)** to confirm apoptotic cells and, anti phosphohistone H3 (red) **(A,a,d)** to reveal proliferating cells. OV means otic vesicle. Large arrowheads pointed to the stream of vagal NCC. White boxes include the region that is shown in close up in the insert. Small white arrowheads point to the proliferating cells. **(B)** The NCC proliferation in the ctrl group is approximately 2.3 fold the H3^+^ cells of BMP2b MO group. The NCC apoptosis in the ctrl group is approximately 0.87 fold the Caspase3^+^ cells of BMP2b MO group. Values are given as mean ± SE. *n* = 6, *t*-test, ^∗^*P* < 0.05, compared with BMP2b MO group; ^#^*P* > 0.05, compared with BMP2b MO group. Bar, 100 μm.

To identify the mechanism by which BMP2b regulates the development of ENS NCCs, we assessed whether a decrease in proliferation or an increase in apoptosis occurs in treated embryos. We first identified the expression of activated caspase 3 in vagal ENS precursors in control and experimental embryos at 30 hpf. Given that GFP is expressed in vagal ENS precursors in *Tg (foxd3: gfp)* embryos, these embryos were used for these studies. No increase in apoptosis was observed in BMP2b MO treated embryos (Figures 5A,f,h), indicating that apoptosis is not the main cause of the loss of ENS precursors in BMP2b MO-treated embryos.

We next studied the pattern of an anti-phosphohistone H3 antibody immunoreactivity in vagal ENS precursors in the BMP2b morphants. BMP2b MO-treated and control *Tg(foxd3:gfp)* transgenic embryos were stained to identify proliferating cells, and the number of proliferating vagal ENS precursors was significantly decreased in BMP2b morphant embryos compared with controls (Figures 5A,b,d). Only 10.8 ± 2.3% of vagal ENS precursors were proliferative in BMP2b morphant embryos vs. 32.2 ± 6.1% in control embryos (*n* = 5, *t*-test, *P* < 0.01, Figure 5B). This result indicates that reduced proliferation in the vagal NCC represents one of the reasons for the loss of ENS precursors.

In conclusion, losing the function of BMP2b results in decreased proliferation of vagal ENS precursors but not increased cell apoptosis.

#### Neuronal Differentiation Is Reduced in BMP2b MO Embryos

**FIGURE 6:**
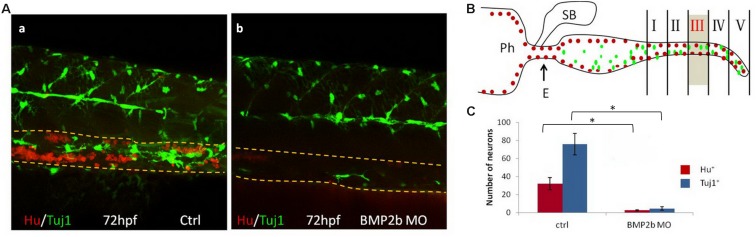
BMP2 function are required for differentiation of neurons. **(A)** Lateral views of the vagal region of 72 hpf wild type(15/15, 100%) **(A,a)** and BMP2b MO treated(32/39, 82.1%) **(A,b)**. Embryos were immunocytochemically double stained with anti Hu antibody (red) and anti Tuj1 antibody (green) (Yellow dashed lines indicate the gut). **(B)** Partition diagram of zebrafish gut. The gut was divided into five areas, the number of neurons of section III were counted (Sb, swim bladder; Ph, pharynx; E, esophagus). **(C)** The number of neurons was counted in different groups. The number of neurons in the ctrl group is approximately 7.8 fold the Hu^+^ cells of BMP2b MO group. The number of neurons in the ctrl group is approximately 14.6 fold the Tuj1^+^ cells of BMP2b MO group. Values are given as mean ± SE. *n* = 6, *t*-test, ^∗^*P* < 0.01, compared with ctrl group.

To further investigate neuron differentiation in the intestines, Hu and Tuj1 (a marker of mature neurons) were used to double-label mature intestinal neurons. Numerous clustered Hu^+^ cells and differentiated Tuj1^+^ cells were noted in the control embryos(Figure 6A,a). In contrast, only a few Hu^+^ and Tuj1^+^ cells were noted in the intestines of the BMP2b-MO embryos (Figures 6A,b,B,C), indicating that neuronal differentiation in the intestinal region is also regulated by BMP2b.

#### GDNF Expression Is Perturbed in BMP2b MO Embryos

**FIGURE 7:**
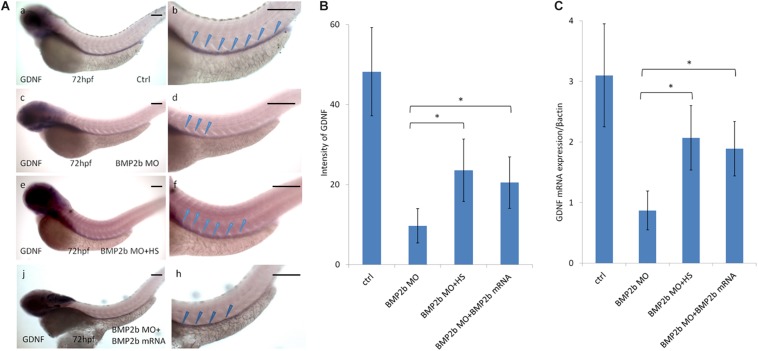
Expression of GDNF in the intestinal mesenchyme requires BMP2 signaling. **(A,a,b)** control embryos(97/103, 94.2%), **(A,c,d)** BMP 2b MO treated embryos) lateral views of 72 hpf whole mount embryos(112/124, 90.3%) that have been hybridized with a gdnf antisense probe. Compared with the control group, there is only partial expression of GDNF at the anterior end of the intestine at this age. **(A,e,f)** BMP2b MO plus heat-shocked at 10 hpf(84/105, 80%), the expression of GDNF was partly rescued. **(A,j,h)** BMP2b MO plus BMP2b mRNA were co-injected, the expression of GDNF was partly rescued(84/105, 80%). **(B)** The intensity of GDNF expression in different group. The expression of GDNF mRNA in the BMP2b MO group is approximately 2.7 fold the intensity of BMP2b MO + HS group. The expression of GDNF mRNA in the BMP2b MO group is approximately 2.5 fold the intensity of BMP2b MO + BMP2b mRNA group. Values are given as mean ± SE. *n* = 6, *t*-test, ^∗^*P* < 0.05, compared with BMP2b MO + HS group or BMP2b MO + BMP2b mRNA group.) **(C)** Real time PCR analysis of GDNF mRNA expression. The GDNF mRNA expression was rescued in BMP2b + HS group, *n* = 6, *t*-test, ^∗^*P* < 0.05, compared with BMP2b MO group. Bar, 100 μm.

In a previous study, GDNF was reported to be a key chemoattractant for ENS NCCs *in vitro* and to regulate ENS NCC proliferation (Gianino et al., 2003). Furthermore, knockdown of GDNF and its receptor disrupted ENS NCC migration (Shepherd et al., 2001). To identify whether the defects in ENS precursor migration in BMP2b MO-treated embryos were related to changes in GDNF expression, we first assessed GDNF expression in BMP2b MO-treated embryos and found that GDNF expression was greatly reduced at the posterior end of the intestine at 72 hpf in the BMP2b morphants (Figures 7A,c,d). By contrast, control embryos exhibited normal GDNF expression at the posterior end of the intestines at this stage (Figures 7A,a,b). Furthermore, we injected BMP2b MO into the *Hsp70: bmp2b-GFP* transgenic embryos at 1-4 cell stage, heat shocked the embryos at 10hpf and then checked the expression of GDNF at 72hpf. The GDNF expression in BMP2b MO-treated embryos could be rescued by heat shock, which also induced the expression of bmp2b mRNA in *Hsp70: bmp2b-GFP* transgenic embryos (Figures 7A,e,f,B,C). These data suggest that BMP signaling is specifically required for GDNF expression in the zebrafish intestine.

#### Rescue of the BMP2-MO and GDNF-MO Phenotype Using *Tg (hsp: BMP2b-GFP)*, bmp2b mRNA, or GDNF mRNA

**FIGURE 8:**
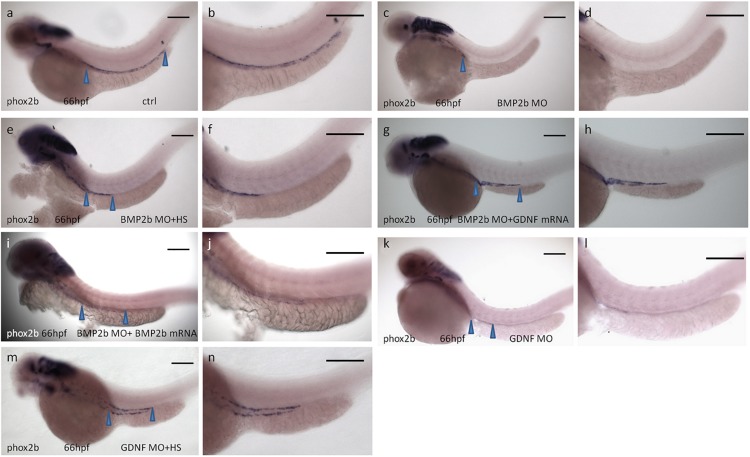
Rescue of BMP2-MO and GDNF-MO phenotypes by heat shock induced BMP2 using Tg(Hsp70:bmp2b-GFP) embryos, as well as by injecting BMP2b mRNA or GDNF mRNA. **(a,b)** Representative image of 66 hpf embryos obtained from control injection(78/84, 92.8%), **(c,d)** injection with BMP2b-MO alone(115/151, 76.2%) or **(e,f)** with BMP2b-MO plus heat-shocked at 10 hpf(120/146, 82.2%) or **(g,h)** with GDNF mRNA(110/153, 71.9%) or **(i,j)** with BMP2b mRNA (63/76, 82.9%), **(k,l)** injection with GDNF-MO alone(115/138) or **(m,n)** with GDNF-MO plus heat-shocked at 10 hpf(105/125, 84%). Arrowheads indicate phox2b expressing in the intestine. Bar, 100 μm.

Our data suggested that the expression of phox2b along the posterior axis of the zebrafish intestine disappeared in BMP2b-MO treated embryos (Figures 4A,d,f). To further corroborate the role of BMP2 in the phenotypic alterations observed in BMP2b-MO treated embryos, rescue experiments were performed. We first micro-injected transgenic zebrafish *Tg (hsp: BMP2b-GFP)* eggs at the one- to two-cell stage with the control MO (control, CTRL) or BMP2b-MO and then subjected them to heat-shock treatment at 10 hpf. We also co-injected BMP2b-MO with BMP2b mRNA. Then we analyzed phox2b expression in the embryos at 66 hpf in each case. ISH images showing the embryos at 66 hpf revealed that phox2b-expressing cells almost disappeared in the intestines of BMP2b-MO treated embryos (Figures 8c,d), while in the embryos treated with BMP2b-MO and heat shocked (Figures 8e,f) phox2b-expressing cells populated the length of the intestine. In addition, BMP2b mRNA injection also partially rescued the phenotype in BMP2b-MO treated embryos (Figures 8i,j). These data suggested that BMP2b was specifically involved in ENS migration and development. Since our data also showed that BMP2b was required for the normal expression of GDNF in the intestine, we continued to evaluate whether GDNF mediates the role of BMP2b during ENS development. First, we downregulated the function of GDNF by injecting the GDNF-MO into the embryos, and we found that the number of phox2b expressing cells was decreased greatly compared with the control morphants (Figures 8k,l), implying that GDNF may be downstream of BMP2b signaling in the regulation of ENS development. Furthermore, we co-injected BMP2b-MO and exogenous GDNF mRNA into the embryos to analyze whether the restoration of GDNF expression in BMP2b-MO treated embryos could rescue phox2b expression, and the result suggested that exogenous GDNF mRNA partially rescued the expression of phox2b (Figures 8g,h).

The above rescue experiments indicated that GDNF truly lies downstream of BMP2 signaling and mediates the role of BMP2 in ENS development. While detailed analysis suggested that the phenotype of GDNF-MO embryos was not as strong as that of BMP2b-MO embryos (Figures 8c,d,k,l), we cannot exclude the possibility that other downstream factors involved in BMP2 signaling are also involved in this process. To examine this hypothesis, transgenic *Tg (hsp: BMP2b-GFP)* zebrafish eggs were injected with GDNF-MO and then subjected to heat shock at 10 hpf, and the expression of phox2b was evaluated at 66 hpf. The results suggested that exogenous BMP2b mRNA could also partially rescue the phenotype in GDNF-MO injected embryos (Figures 8m,n).

In conclusion, these data indicated that BMP2b is involved in ENS development and that this process is mediated, at least in part, by GDNF signaling in zebrafish.

## Discussion

The purpose of this study was to identify the role of BMP signaling in the ENS defect observed in HSCR patients and the detailed role of BMP in ENS development *in vivo*. We first demonstrated that BMP2 expression was downregulated in HSCR. Our data also demonstrated the role of BMP2 in the proliferation, migration and differentiation of primary NCCs *in vitro*. Second, we investigated the expression of BMP2b, BMPR1a and BMPR1b in the developing zebrafish gut. BMP2b and BMPR1a were strongly expressed during the early development of the ENS. The location and timing of these expression patterns support a potential role of BMP signaling in ENS development *in vivo*.

In mice, BMP2 expression correlated with NCC production exhibits a spatiotemporally dynamic pattern in the surface ectoderm adjacent to the neuroectoderm (Ohnemus et al., 2002). Interference with BMP2 signaling in the dorsal neural tube results in an absence of NCC migration (Kanzler et al., 2000; Ohnemus et al., 2002). Furthermore, in BMP2-mutant embryos, Crabp1 reactivity cannot be detected, and its absence is correlated with an absence of branchial arches (Correia et al., 2007). More recent mouse studies demonstrated that NCCs could be induced in BMP2 mutants and that the absence of migratory NCCs in these mutants does not derive from increased death of NCCs; thus, it is likely that BMP2 is required to trigger the migration of NCC progenitors (Kanzler et al., 2000). Therefore, the results we obtained were different to those from previous studies. In our study, we first demonstrated that BMP2 promoted the proliferation, migration and differentiation of primary NCCs (Figure 3). We further found that GDNF exhibited a more profound effect on inducing migration and differentiation compared with BMP2 alone under similar culture conditions (Figures 3B,C). In addition, our data demonstrated that BMP2 increased the number of nNOS- and TH-expressing neurons *in vitro* (Figure 3D), and these results were consistent with *in vivo* studies performed in mice (Stull et al., 2001; Reiriz et al., 2015). BMP2-induced signal transduction is associated with the phosphorylation and nuclear translocation of receptor-activated Smad proteins. In Smad-dependent signaling, phosphorylated R-Smad (Smad1, 5, or 8) complexes with Smad4 and co-translocates into nuclei to regulate gene expression (Wrana, 2000). We further demonstrated that the level of pSmad1 was reduced, while that of pSmad5/8 was not in the spasm segment in HSCR (Supplementary Figure S2). Our data suggested that BMP2-Smad1 signaling is involved in the molecular mechanism of HSCR.

There are two zebrafish BMP2 orthologs: BMP2a and BMP2b. Schumacher et al. (2011) proposed a swr/bmp2b mutant zebrafish line, which shows the loss of neural crest progenitor cells. Previous research demonstrated that the expression domains of bmp2b and bmp7 in the posterior tail likely mediate the dorsalization of neural tissue at postgastrulation stages (Nguyen et al., 2000). Furthermore, in this study, wild-type zebrafish and several transgenic zebrafish lines were used to investigate the effect of BMP2 on ENS development *in vivo*. Our data demonstrated the loss of ENS precursors in the BMP2b signaling pathway defected (Figures 4A,C) and increased ENS NCCs in BMP2b-overexpressing embryos (Figure 4D). Antagonizing BMP2 activity in the cranial neural crest in transgenic mice also results in the inhibition of NCC migration from targeted areas. Moreover, the absence of neural crest migration correlates with phenotypic features expected to arise from neural crest deficiencies (Anderson et al., 2006). The results indicate that the antiapoptotic effect of BMP2 is restricted to the neural crest population. Furthermore, when the expression of BMP2b was downregulated, we did not observe an obvious increase in cell apoptosis in the vagal neural crest (Figure 5). These data suggest that apoptosis is not the main mechanism that causes the loss of ENS precursors when BMP2 signaling is perturbed.

We also found that BMP2 is necessary for NCC migration from the vagal region to the anterior end of the intestine in ENS development. This result is significant and is consistent with other studies showing that BMP2 plays a role in determining the pattern of the migration of a number of neural crest populations (Kanzler et al., 2000; Ohnemus et al., 2002; Ragland and Raible, 2004; Correia et al., 2007). Our results further demonstrated that BMP2 regulates vagal NCC migration, and ENS defects in BMP2b MO-treated embryos appear to arise as a result of a reduced number of ENS precursors generated within the vagal NCC when BMP2 signaling is inhibited.

A previous study demonstrated that BMP2 acts as a mitogen in ENS neurospheres and NCC migration in chicks (Goldstein et al., 2005) and mice (Fu et al., 2006), suggesting that the intestinal and ENS patterns are defective when BMP signaling is perturbed. Here, we demonstrate that overexpression results in similar mitogenic activity of BMP2b *in vivo*. The possibility that BMP2b activity may represent a direct effect of or be attributed to the increased secretion of other mitogens from the intestinal mesenchyme remains unverified. Pyati et al. (2006) found that BMP signaling is required for zebrafish cloaca development. A previous study in chicks indicated that BMP activity is required for ENS and visceral smooth muscle development (Barbara et al., 2005). Our data also indicate that inhibition of BMP2b signaling results in the failure of intestinal smooth muscle differentiation in the gut of zebrafish (Figure 4B), and these factors may be involved in intestinal smooth muscle differentiation. All of these results suggest that BMP signaling is required for the development of multiple types of tissues in the gut and plays a novel role in the interactions and reciprocal communications of these tissue layers.

Multiple factors promote migration by increasing cell proliferation or via a direct effect on migration. GDNF is critical for stimulating the migratory behavior of these precursors along the intestine (Shepherd et al., 2001; Gianino et al., 2003). We found that BMP2 alone is not sufficient to promote the migration of NCCs from the intestine *in vitro*. Our data in zebrafish are also consistent with data indicating that BMP2b regulates GDNF activity in ENS development given that perturbation of BMP2b results in a loss of GDNF expression in the intestinal mesenchyme (Figures 7, 8). In our *in vivo* experiment, two rescue experiments showed that BMP2b specifically regulated ENS development: BMP2b mRNA injection rescue experiment and “heat shock” induced BMP2b mRNA rescue experiment. Our results showed that the rescue efficiency of injected BMP2b mRNA was lower than the efficiency of heat shock induced BMP2b mRNA. The possible underlying reason is that the injected mRNA couldn’t exist in the embryos as long as late heat shock induced BMP2b mRNA could. All in all, based on our data, we conclude that BMP2 and GDNF function together in crest cell migration. Although the mechanism by which GDNF promotes migration and the interaction between BMP2 and GDNF remains unknown, our data indicate that GDNF, as one of the factors, is located downstream of BMP2 signaling.

Brewer et al. (2005) investigated the pattern of BMP2 and the BMP receptor in the fetal gut of humans, demonstrating that BMP2 expression is initially limited to the epithelium, but is subsequently observed in the myenteric plexus. Interestingly, BMPR1a/1b is strongly expressed throughout the stage of ENS development (Brewer et al., 2005). Our data show that the expression of BMP2b and BMPR1a is consistent with ENS development (Supplementary Figure S5). These data suggest that BMP2 and BMPR1 are required for ENS development in the intestine. The pleiotropic effects of BMPs in the development of the ENS are well characterized (Alcmène and Kessler, 2012), but whether aberrant BMP activity is involved in the regulation of ENS defects in patients is largely unknown. Wu et al. (2014) found that the BMP2 level was increased in the stenotic segments of individuals with HSCR compared with normal segments, suggesting that a low concentration of BMP2 promotes ENS development. In contrast, most previous studies have demonstrated that BMP signaling is activated in the gut of all the tissue layers during development (Anderson et al., 2006; Schumacher et al., 2011). In addition, although the roles of BMP in ENS development have been reported, the underlying mechanism remains to be determined, especially in HSCR. To study the effect of BMP signaling on ENS development in HSCR, we investigated BMP2 and GDNF expression in different segments of the colon in individuals with HSCR and normal individuals (Figure 1). BMP2 was abundantly expressed in the colon in both the HSCR and normal groups, indicating that BMP2 is required for ENS development and is related to the etiology of HSCR. BMP2 is involved in regulating GDNF expression in the intestine, which highlights the central role of the BMP-GDNF cascade in intestinal patterning and ENS. There is a fine balance between BMP signaling pathways and GDNF in ENS development and perturbation of this balance will lead to intestinal patterning defects and intestinal aganglionosis.

## Conclusion

Our results support the essential role of BMP2 signaling in the development of HSCR *in vitro* and *in vivo*. We observed critical outcomes due to abnormal BMP2 signaling. First, the downregulation of BMP2 and GDNF indicated the possible roles of these factors in HSCR, and *in vitro* experiments indicated that BMP2 and GDNF play a crucial role in regulating NCC proliferation, migration and differentiation. Second, in zebrafish, an ENS precursor migration defect in the colonization of the entire gut results in an absence of enteric ganglia along the entire colon due to the loss of function for BMP2 signaling. Third, GDNF is located downstream of BMP2 and mediates the role of BMP2 in the regulation of ENS development. In conclusion, our data indicate that the BMP2-GDNF pathway is involved in ENS development. The pathological changes in BMP2 and GDNF observed in HSCR indicated the possibility that the BMP2-GDNF cascade is essential for the development of HSCR in patients.

## Data Availability Statement

The raw data supporting the conclusions of this manuscript will be made available by the authors, without undue reservation, to any qualified researcher.

## Ethics Statement

The studies involving human participants were reviewed and approved by the Ethical Committee of Psychology (ECP) affiliated with the Chongqing Medical University. Written informed consent to participate in this study was provided by the participants’ legal guardian/next of kin. The animal study was reviewed and approved by the Laboratory Animal – Requirements of Environment and Housing Facilities, Chongqing Administration Rule of Laboratory Animal, Chongqing Medical University.

## Author Contributions

ZG conceived the study and designed the experimental procedures. ZG, SH, LL, YW, and XJ performed the experiments and carried out the data analysis. SL, XL, and XY collected field data. ZG, MY, and SH carried out the statistical analyses and wrote the manuscript. All authors gave final approval for publication.

## Conflict of Interest

The authors declare that the research was conducted in the absence of any commercial or financial relationships that could be construed as a potential conflict of interest.

## Supplementary Material

FIGURE S1 The expression of Smad1 decreased in the spasm segment of HSCR patients. **(A,B)** Western blot analysis of Smad1, Smad5, and Smad8 expression in normal colon and different segments of HSCR. Values are given as mean ± SE. *n* = 3, *t*-test, ^∗^*P* < 0.05, ^#^*P* > 0.05, compared with normal colon and distension segment of HSCR.

FIGURE S2 BMP2 and GDNF promotes the proliferation of NCCs. Proliferation of NCCs were measured using BrdU Stainning. Compared with control group **(A)**, both BMP2 **(B)**, and GDNF **(C)** promote proliferation of NCCs. **(D)** BMP2 and GDNF together caused enhanced proliferation in NCCs. **(E)** The number of BrdU^+^ cells in different group. Values are given as mean ± SE. *n* = 3, *t*-test, ^∗^*P* < 0.05, compared with ctrl group.

FIGURE S3 Progress of enteric precursors through the zebrafish intestine. Crestin and phox2b labels enteric precursors as they migrate through the intestine. Crestin is expressed in precursors at 34 hpf **(A,a)** as they enter the digestive system by migrating along the posterior margin of the pharynx, through the esophagus, and into the anterior intestine. At 52 hpf **(B,a)** precursors travel in two lateral lines on either side of the intestine and have migrated approximately a fourth of the way through intestine. By 56 hpf **(B,b)** precursors occupy a third of theintestine. By 60 hpf **(B,c)** precursors migrate to approximately the midpoint of the intestine and finish migration by 66 hpf **(B,d)**. Bar, 100 μm.

FIGURE S4 The expression pattern of BMP2b and BMPR correlate with the development of ENS. **(A,B,E,F)** Wholemount embryos hybridized *in situ* with a BMP2b antisense probe at the indicated developmental stages. **(C,D,G,H)** Wholemount embryos hybridized *in situ* with a BMPRIa antisense probe at the indicated developmental stages. **(I,J)** Wholemount embryos hybridized *in situ* with a BMPRIb antisense probe at the indicated developmental stages. **(K,L)** 60 hpf wholemount *in situ* hybridized embryos with a phox2b antisense probe to reveal the distribution of the ENS NCC in the intestine. At all stages examined, BMP2b and BMPRI are expressed in regions that correlate with the development of the ENS. Furthermore, comparison of the pattern of BMPRIa expression in the intestine at 60 hpf shows that phox2b expressing ENS NCC is located in the BMPRIa expression domain (^∗^ indicate gut). Bar, 100 μm.

FIGURE S5 Checking the work efficiency of Bmp2b MO and bmp2b mRNA by Western Blot. Line1 and line2 are controls, which indicated the Bmp2b antibody works well. Line3 is Bmp2b MO sample, line4 is bmp2b mRNA injected sample, these two lines indicated the Bmp2b MO and bmp2b mRNA for injection work well.

TABLE S1 Primer sequences for ISH.

TABLE S2 Primer sequences for qRT-PCR.

## Abbreviations

α SMA: α smooth muscle actin
BDNF: brain-derived neurotrophic factor
BMP2: bone morphogenetic protein 2
BMP4: bone morphogenetic protein 4
BMPs: bone morphogenetic proteins
CDs: coding sequence
ChAT: choline acetyl transferase
CNS: central nervous system
CNTF: ciliary neurotrophic Factor
END3: endothelin-3
ENS: enteric nervous system
FCM: flow cytometer
GDNF: glial cell line-derived neurotrophic factor
GFAP: glial fibrillary acidic protein
GFP: green fluorescent protein
HSCR: Hirschsprung’s disease
ISH: *in situ* hybridization
MO: morpholino
NCCs: neural crest cells
NGF: nerve growth factor
nNOS: neuronal nitric oxide synthase
PBS: phosphate buffer solution
PNS: peripheral nervous system
TUJ1: neuronal class III β-tubulin
