# Supplementary figures and images for: BMP2 Is Related to Hirschsprung’s Disease and Required for Enteric Nervous System Development
Source: Front Cell Neurosci. 2019 Dec 3;13:523. doi: 10.3389/fncel.2019.00523 (PMC6901830; doi:10.3389/fncel.2019.00523)

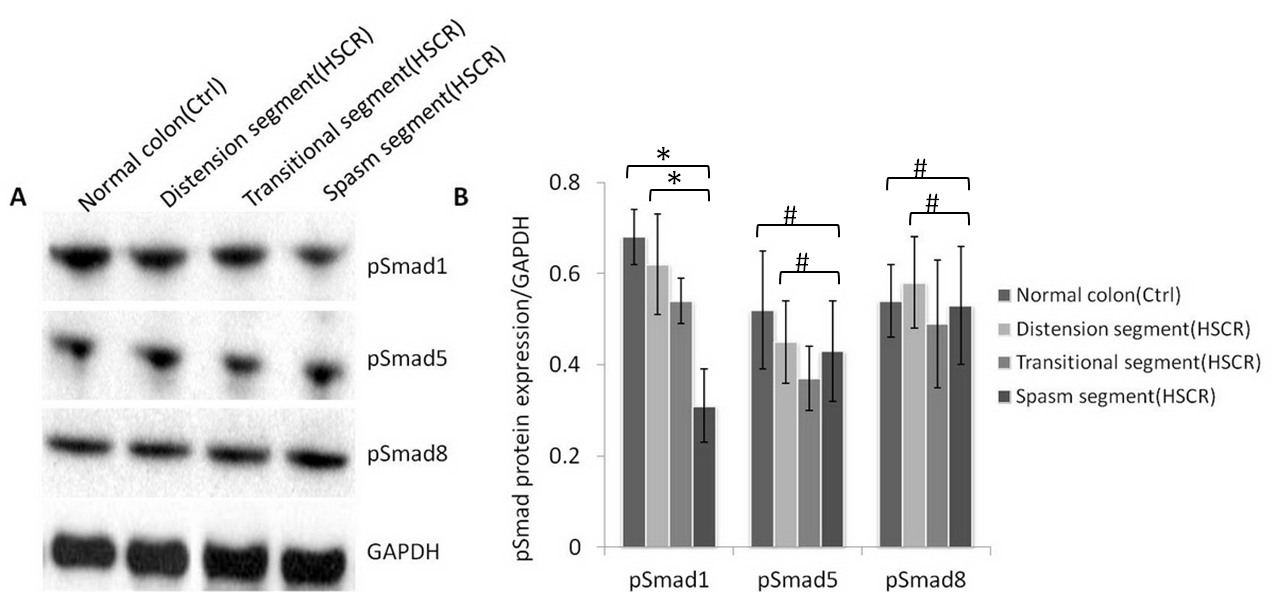

Supplement: FIGURE S1 — The expression of Smad1 decreased in the spasm segment of HSCR patients. (A,B) Western blot analysis of Smad1, Smad5, and Smad8 expression in normal colon and different segments of HSCR. Values are given as mean ± SE. n = 3, t-test, ∗P < 0.05, #P > 0.05, compared with normal colon and distension segment of HSCR. [file Image_1.TIF]

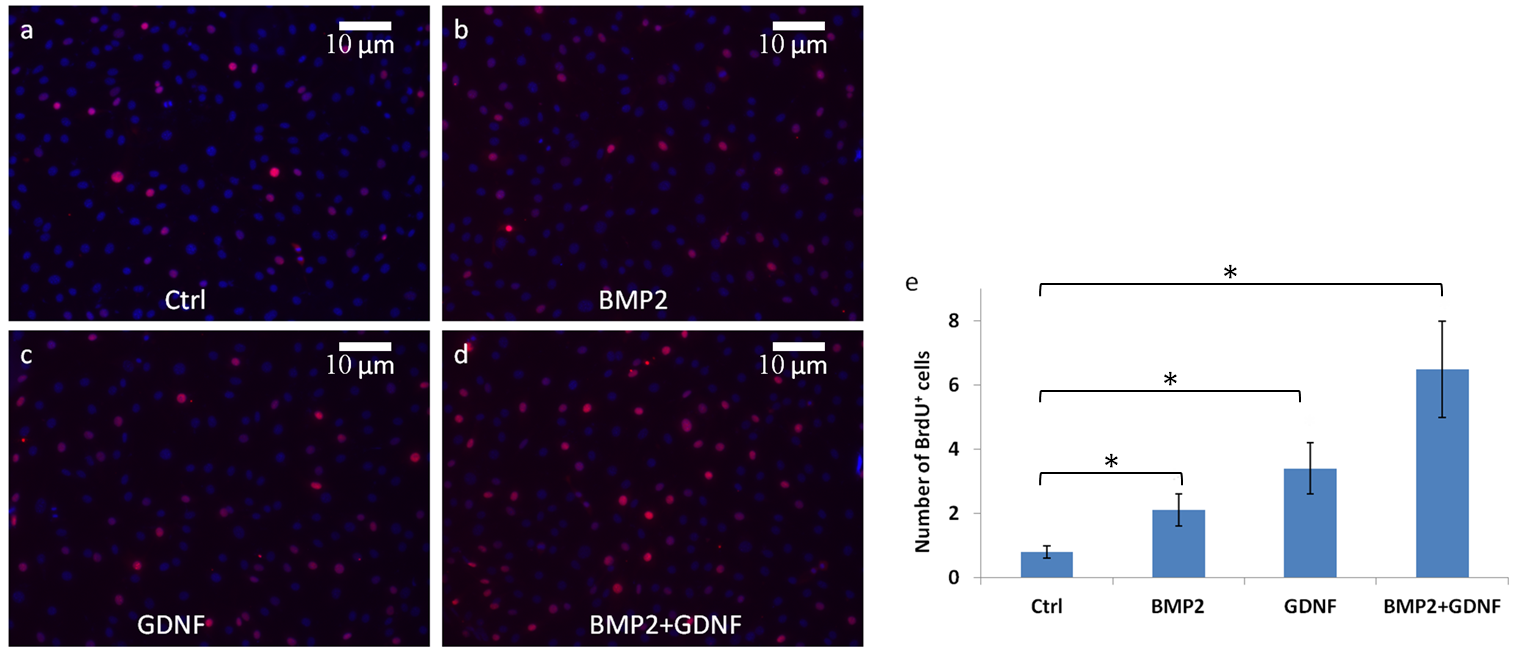

Supplement: FIGURE S2 — BMP2 and GDNF promotes the proliferation of NCCs. Proliferation of NCCs were measured using BrdU Stainning. Compared with control group (A), both BMP2 (B), and GDNF (C) promote proliferation of NCCs. (D) BMP2 and GDNF together caused enhanced proliferation in NCCs. (E) The number of BrdU+ cells in different group. Values are given as mean ± SE. n = 3, t-test, ∗P < 0.05, compared with ctrl group. [file Image_2.TIF]

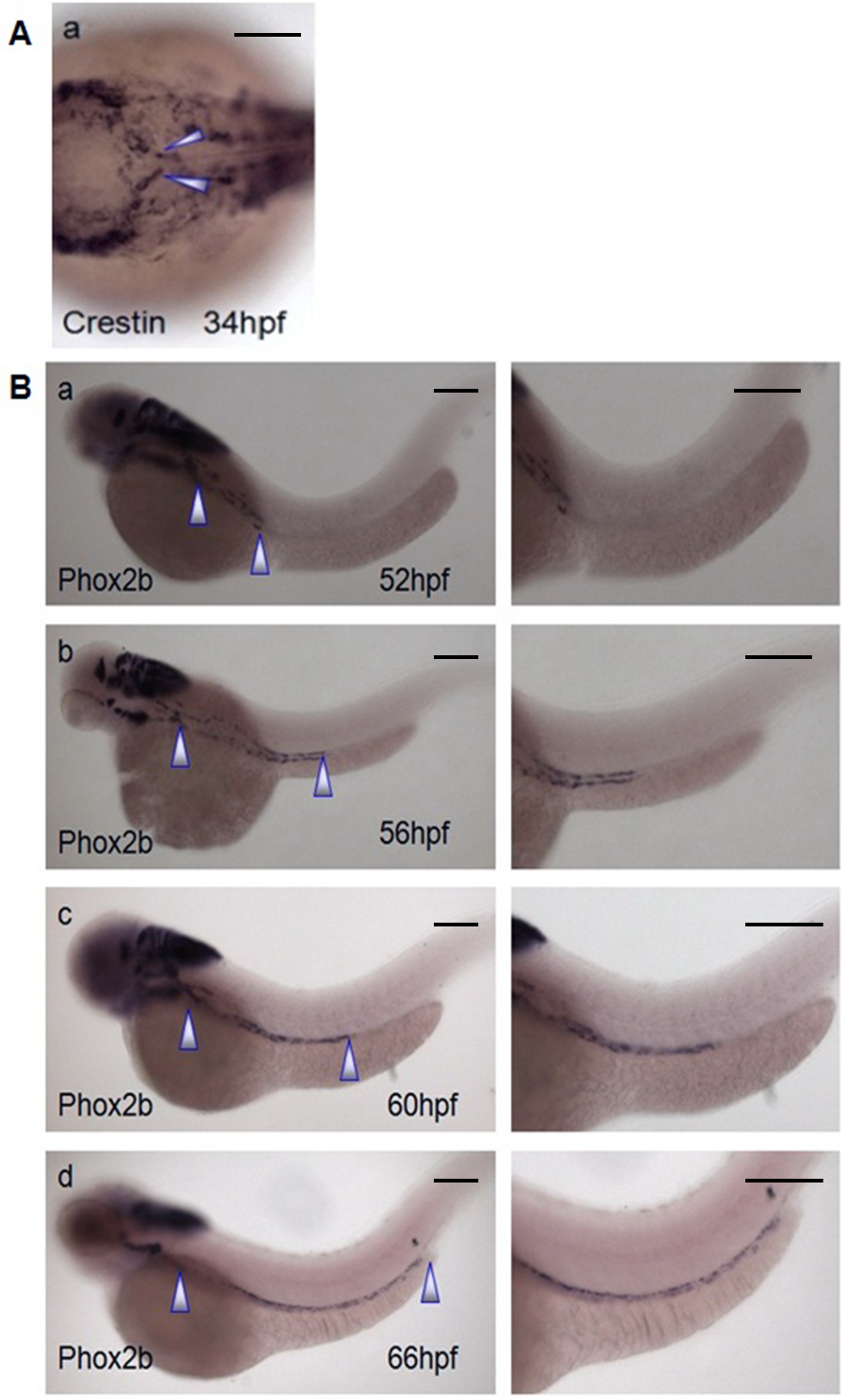

Supplement: FIGURE S3 — Progress of enteric precursors through the zebrafish intestine. Crestin and phox2b labels enteric precursors as they migrate through the intestine. Crestin is expressed in precursors at 34 hpf (A,a) as they enter the digestive system by migrating along the posterior margin of the pharynx, through the esophagus, and into the anterior intestine. At 52 hpf (B,a) precursors travel in two lateral lines on either side of the intestine and have migrated approximately a fourth of the way through intestine. By 56 hpf (B,b) precursors occupy a third of theintestine. By 60 hpf (B,c) precursors migrate to approximately the midpoint of the intestine and finish migration by 66 hpf (B,d). Bar, 100 μm. [file Image_3.TIF]

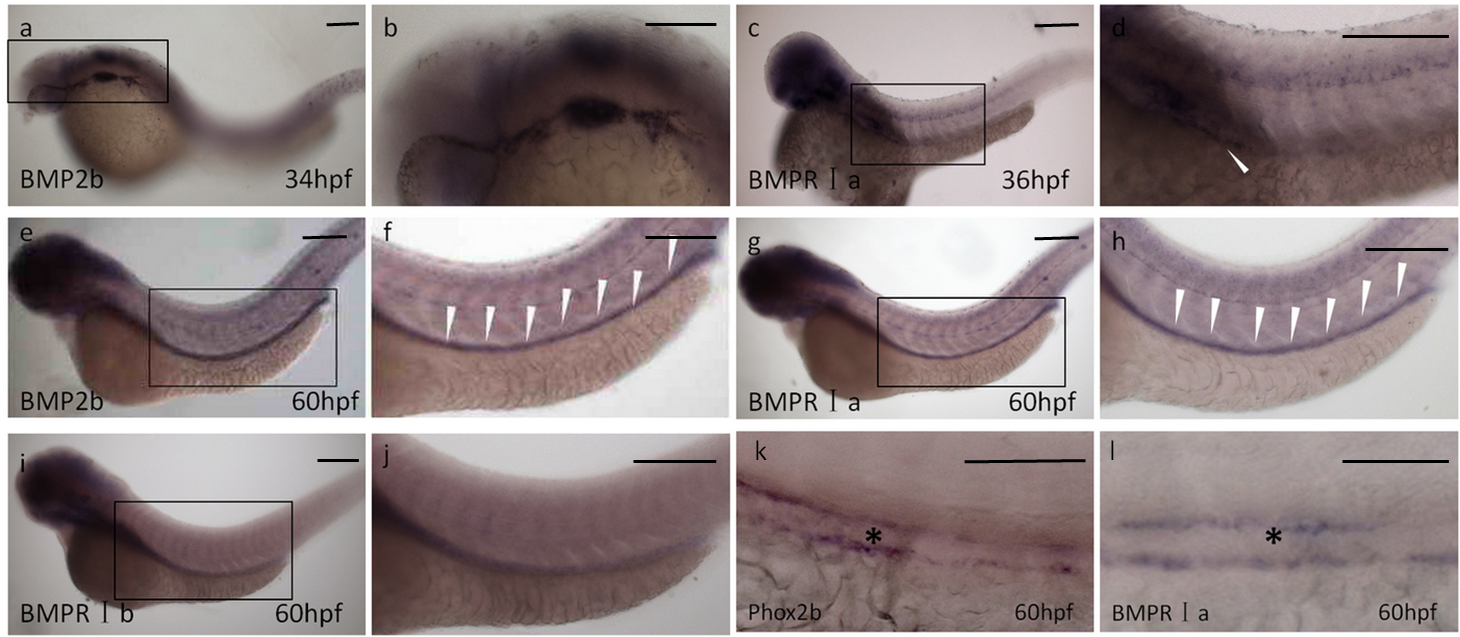

Supplement: FIGURE S4 — The expression pattern of BMP2b and BMPR correlate with the development of ENS. (A,B,E,F) Wholemount embryos hybridized in situ with a BMP2b antisense probe at the indicated developmental stages. (C,D,G,H) Wholemount embryos hybridized in situ with a BMPRIa antisense probe at the indicated developmental stages. (I,J) Wholemount embryos hybridized in situ with a BMPRIb antisense probe at the indicated developmental stages. (K,L) 60 hpf wholemount in situ hybridized embryos with a phox2b antisense probe to reveal the distribution of the ENS NCC in the intestine. At all stages examined, BMP2b and BMPRI are expressed in regions that correlate with the development of the ENS. Furthermore, comparison of the pattern of BMPRIa expression in the intestine at 60 hpf shows that phox2b expressing ENS NCC is located in the BMPRIa expression domain (∗ indicate gut). Bar, 100 μm. [file Image_4.TIF]

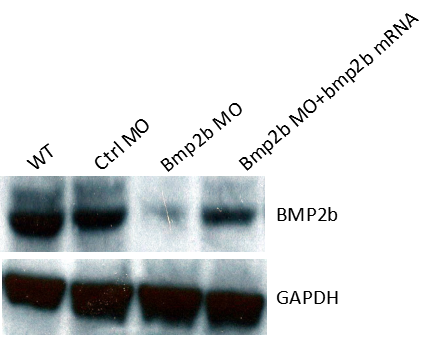

Supplement: FIGURE S5 — Checking the work efficiency of Bmp2b MO and bmp2b mRNA by Western Blot. Line1 and line2 are controls, which indicated the Bmp2b antibody works well. Line3 is Bmp2b MO sample, line4 is bmp2b mRNA injected sample, these two lines indicated the Bmp2b MO and bmp2b mRNA for injection work well. [file Image_5.TIF]
