# Supplementary material for: BMP2 Is Related to Hirschsprung’s Disease and Required for Enteric Nervous System Development
Source: Front Cell Neurosci. 2019 Dec 3;13:523. doi: 10.3389/fncel.2019.00523 (PMC6901830; doi:10.3389/fncel.2019.00523)
Supplement: TABLE S1 — Primer sequences for ISH. [file Table_1.docx]

**Table. S1. Primer sequences for ISH**

| Name | Forward sequence | Reverse sequence | Length (bp) |
| --- | --- | --- | --- |
| Phox2b | CTCGCGAGTCAGATTTCTTCCCAGTGC | AGTACAACCCAATCAGGACGACGTTTGG | 392 |
| crestin | GAGAAGCCCTCATCAGAGAGTTTG | TAATACGACTCACTATAGGGGTTGCTTGTCAGGCAGAATCAGG | 825 |
| BMP2b | CTGATCATGGTCGCCGTGGTC | GAGATTGTTCTCATCGGCAACCGC | 1252 |
| GDNF | GTCTAAAATGAAATTATGGGACATTC | TGCAACGCAAGCACACTTTTTAGCGG | 715 |
| αSMA | ACAGGGGAAGATGTTGAGT | GCTGTGAGAGGAGTTGGT | 832 |
| BMPR1a | CCC GAT GAT GCC AAG AAC AAT A | TCA GCA GCG ATA AAG CCG AGT A | 668 |
| BMPR1b | GTC GGG AGA CAG GAA GTG C | CGA GAC GGA ATA GGC AAG T | 884 |
