# Supplementary material for: BMP2 Is Related to Hirschsprung’s Disease and Required for Enteric Nervous System Development
Source: Front Cell Neurosci. 2019 Dec 3;13:523. doi: 10.3389/fncel.2019.00523 (PMC6901830; doi:10.3389/fncel.2019.00523)
Supplement: TABLE S2 — Primer sequences for qRT-PCR. [file Table_2.docx]

**Table. S2.** **Primer sequences for qRT-PCR.**

| Name | Forward sequence | Reverse sequence |
| --- | --- | --- |
| BMP2 | ACC CGC TGT CTT CTA GCG T | TTT CAG GCC GAA CAT GCT GAG |
| GDNF | GGC AGT GCT TCC TAG AAG AGA | AAG ACA CAA CCC CGG TTT TTG |
| Nnos | GGG GCT CAA ATG GTA TGG | GAT GAA GGA CTC CGT GGC |
| TH | CCA AGG TTC ATT GGA CGG C | CTC TCC TCG AAT ACC ACA GCC |
| ChAT | GGC CAT TGT GAA GCG GTT TG | GCC AGG CGG TTG TTT AGA TAC A |
| Tuj1 | CCACCTTCATTGGCAACAGC | GCTTCCGATTCCTCGTCATCA |
